# Investigation of empty container shortage based on SWARA-ARAS methods in the COVID-19 era

**DOI:** 10.1186/s12544-022-00531-8

**Published:** 2022-03-21

**Authors:** Arda Toygar, Umut Yildirim, Gani Mustafa İnegöl

**Affiliations:** 1 Artvin Coruh University, Maritime and Port Management Program, Artvin, Turkey; 2 Karadeniz Technical University, Maritime Transportation and Management Engineering, Trabzon, Turkey; 3 Kocaeli University, Department of Motor Vehicles and Transportation Technologies, Kocaeli, Turkey

**Keywords:** Maritime transport, COVID-19, Container shortage, SWARA, ARAS

## Abstract

A shortage of empty containers has become a global crisis with more devastating effects than during previous periods when combined with various problems arising from the COVID-19, such as an increase in an imbalance of global trade between supply and demand, a decrease in the workforce, and restrictions by countries or regional quarantine practices. The absence of empty containers in regions where they are needed slows down industrial activities and locks the global supply networks, necessitating the use of alternative methods that are inefficient. Although this shortage causes many disruptions in global trade, solutions to the issue have not been studied in detail. Therefore, the aim of this study was to determine the issues caused by the shortage of empty containers and to rank the appropriate solutions. Four main criteria and sixteen subcategories used to define the issues, as well as a multi criteria decision model comprising five criteria for the solutions, were proposed based on information from the literature, sectorial publications, and expert opinions. The issues’ weighted order of importance in our proposed model was calculated using the SWARA (Step-wise Weight Assessment Ratio Analysis) method; solutions were ranked using the ARAS (Additive Ratio Assessment) method. The results of the study revealed that the issues were ranked in importance as cost increases, uncertainty in the supply chain, volume loss, and increases in blank sailing announcements. Appropriate solutions were ranked as booking guarantee applications and information communication technologies, using shipper-owned containers, inducement calls, and E2E (end to end) delivery services.

## Introduction

The share of container uses within the global commodity trade industry, which has helped innovating international trade, has increased each year. In fact, in 1990, the global usage was 28.7 million TEUs (twenty-foot equivalent units) and increased to 815.6 million TEUs in 2020 [1, 2]. One important reason for the high demand for containers used in transportation is that containers play a key role in ensuring a low-cost global trade [3]. Although the shipping charge was $5.83/T during the precontainer period, this decreased to $0.158/T after containers were introduced into the maritime industry [4]. In spite of such important advantages within the global trade industry, a container shortage is an issue that negatively affects the transport chain within this industry. Although this is not a new issue, it has been widely studied. There are various factors that have resulted in an unpredictable, short-term empty-container shortage, such as weather conditions, strikes at the ports, demand uncertainty, and the lack of suitable container type for export cargo [5–7]. Although many strategies have been developed to resolve the issues, the pandemic from the COVID-19 has been the biggest global crises that has had devastating effects on both the economy and health [8, 9].

Important factors that determine delivery times for container transportation include the number of ships providing service to ports of call, delivery of full containers to the consignee after unloading them in the ports of importation, and transfer of the empty containers to the region where needed [10, 11]. The restrictions imposed and quarantine decisions made by the authorities to reduce the spread of COVID-19 have increased delivery times by directly affecting the workforce, and the containers are being kept for longer periods in the port areas, warehouses, or shipboards [12]. Moreover, these decisions made by national and international authorities increase ship traffic in the forelands and the charge carrier density in the hinterlands, causing congestion at the ports and longer shipping times [13]. Shipping lines providing services to the regions in which quarantine decisions were made can add different ports of call to their shipping schedules by announcing “blank sailing” to ports within the region until the port congestion problem is eliminated [14]. For example, Youd [15] has discussed the increased delays in delivering cargoes because of heavy ship traffic at the ports, and has indicated that the shipping lines prefer ports that have less ship traffic rather than large ports as discharge ports. These practices led to restricted global-freight mobility, the inability to move empty containers from the demand point to the supply point, and the inefficient use of global container capacity.

The empty containers needed to deliver available-to-promise cargoes to importing companies could not be positioned at the necessary points, which caused significant uncertainties in commercial activities and relationships for both consumption and industrial markets. Accordingly, companies could not make raw materials and or plan production and dealt with unforeseen financial losses in supply chain processes, such as packaging, storage, and distribution, as a result of uncertain delivery times. Thus, it was very important to examine the issues within the container transport chains and develop strategies by which to resolve the issues by incorporating many companies into the process; however, it was observed that the effects of COVID-19 on transportation processes were not completely understood, and many precautionary strategies developed during the previous periods for the survival of companies were insufficient [16–18]. The present study analyzed the negative effects of the container shortage on global trade during the COVID-19 period and the most appropriate methods proposed by which to resolve these issues by using input from experts working in beneficial cargo owner (BCO) and freight forwarder (FFW) businesses that represent the demand side of the industry. We believe that the results of the present study will contribute the following three important issues to the literature: (1) the issues created by the container shortage, which existed in the past, but increased with the devastating effects of COVID-19 on global trade, were identified; (2) the most effective methods by which to resolved the issues using data taken from industry stakeholders were examined; and (3) suggestions to increase the resilience of the sector were presented.

## Literature

### Problems responsible for causing the empty container shortage during the COVID-19 pandemic

In container transportation operations, uncertainty in demand can be experienced periodically. Maritime companies have developed several of operational and strategic practices to deal with these situations when they occur, including omitting some ports in the itineraries or the blank sailing of all ports in the itineraries and assigning ship to schedules on different routes [18]. These practices used as solutions by maritime companies can, however, result in empty containers failing to be positioned at the necessary points [19] and thus lead to redundant storage of containers in regions where importation is high, as well as to container shortages in regions where exportation is high [20]. This issue was addressed in a study conducted by Ko [21], where it was highlighted that in countries with high global trade volumes, there is considerable demand for empty containers, and when the demand cannot be met, the available-to-promise cargoes are kept in warehouses. Empty containers that are rendered out of use by keeping them in warehouses or ports cause both cost and time losses for maritime companies, as the storage cost of these containers is almost equivalent to that of full containers. Moreover, when including the use of operational equipment and personnel costs to ship these containers, the total cost can run much higher even [22].

Seven of the world’s 10 largest container ports are located in China, and more than 50% of the global container shipping volume is handled at Chinese ports, the main reason being that China is not only the world’s largest exporter but also the world’s second-largest importing country. This global structure governing exports and imports necessitates the formation of a China-oriented container positioning cycle in container transportation. After a full container is discharged according to this positioning cycle, the container needs to be quickly positioned in China, regardless of whether it is empty or full and then shipped to different regions once it is full. This cycle plan increases the number of containers exported from China and leads to an insufficient number of imported containers. In addition, trade volumes of containerized cargoes increase rapidly with the increase in China-based trade activity that typically occurs before the New Year [14]. The empty container shortage recently experienced can be attributed to all the commercial activities carried out in this process [22]. The emergence of COVID-19 in China, which is recognized as the export center of the world, during the period of the highest export volumes resulted in a rapid decrease of commercial activity in the country and a much more intensive container shortage problem in the world than ever seen in the past [23]. This problem was analyzed by Xu [24] with a regression model using data obtained from 14 different Chinese ports. The results of their study revealed that the port operation processes for both export and import cargoes in China were adversely affected by COVID-19, being responsible for a 20–50% decrease in cargo volumes at Chinese ports [25].

After the declaration of COVID-19 as a pandemic, global-scale quarantine and restrictive practices were applied all around the world. These practices have also led to disruptions in global-scale supply chain networks [26], as the national and international quarantine and restriction policies imposed in the first half of 2020 have resulted in many countries experiencing foreign exchange and labor shortages and sharp decreases in consumer demand [27]. Within this period, 255 million full-time employees working in global-scale operations have been laid off [28]. The most intensive impact of the pandemic on global trade volumes was observed in the second quarter of 2020, which witnessed a 21% decrease in value-based global export and import volumes [29]. More than 80% of international trade is carried out by maritime transport [2], a global trade figure that suggests a problem in maritime transport will also have an impact on global trade. Indeed, in a study by Verschuur et al. [30] conducted using ship monitoring data, it was concluded that volume losses of between 206 and 286 million tons in global maritime trade occurred in the first eight months of 2020. When shipping companies do not get sufficient bookings from ports in their regions of operation due to decreased trade volumes they reduce their port calls or declare blank sailing until the demand in the region reaches desired levels. In the second quarter of 2020, there was a 17% decrease in the number of ports of call [31], and in the first half of 2020, the number of ships actively operating decreased by 69% compared to that of 2017 [32]. In a study conducted by Narasimha et al. [33] that compared quantitative performance data from the COVID-19 and pre-COVID-19 period, it was determined that there was a decrease in cargo volumes and the number of ports of call. In the same study, the effects of COVID-19 on maritime operations were examined using the data obtained from 87 maritime industry experts, where the results showed that the three most common problems were associated with the workforce, the decrease in load volumes, and operational delays, in descending order of magnitude. During this period, it was announced that among three key shipping alliances, there were 126 blank sailings on the Trans-Pacific trade route and 94 on the Asia-Europe route [34]. In the period leading to October 2020, there were 515 blank sailings [35]. In the Suez Canal, one of the most important transit routes on the Far Eastern Europe route, the number of transits in May decreased by 32% to reach an all-time low [36]. In 2021, shipping companies announced “blank sailing” for 919 container ships on Transpacific and Asia-Europe routes and decided to temporarily suspend the port calls of the ships [37]. Container transportation is also defined as regular line transportation because itineraries, price tariffs, and ports of call are determined before the voyage [38]. Most maritime companies publish their schedules, including the ports of call on the service route, months in advance [19], which means that when the itinerary of container transportation is not executed as planned, this decreases the reliability level of maritime companies [39]. Although time losses that affect fixed schedules are experienced due to weather conditions, port planning, and problems in operational processes, these losses are tolerated by customers. However, as of 2020, sharp declines in the reliability of itineraries and problems caused by container shortages have decreased the reliability levels for container line operators considerably. In a report investigating the reliability of itineraries using data obtained from 34 trade routes and more than 60 maritime routes, it was determined that the reliability decreased to 34.9% in the first quarter of 2021, the lowest level since 2011 [40]. During this period, the precautionary strategies applied by the maritime companies were not accepted by the BCO, and complaints were made to many different commissions, such as the FMC (Federal Maritime Commission). A majority of the complaints involved the decreased number of sailings (navigations), blank sailing advertisements, low level of schedule reliability, container rollover, additional costs, and container shortage [35].

The rapid decline in global trade volumes caused by COVID-19 also triggered significant time losses in port operations [33]. This is because the variation in international trade volumes is one of the most important variables that directly affect the efficiency of container ports [24]. Significant disruptions particularly occurred at the entrances and exits of highway and water transportation connections to ports after COVID-19 was declared a pandemic [41]. In addition, the restrictions imposed during this period have brought basic logistics activities, such as inland transport, customs, and warehousing, to a standstill and have caused empty containers to remain at the ports in Europe and America, leading to container shortage problems in other regions [42]. In the first half of 2020, the quarantines imposed at the ports and the limited number of personnel available to provide services increased the costs of container transportation [26]. The impact of all these negative developments can be seen in the China Containerized Freight Index as a 50% increase [43]. In the first three quarters of 2021, 40 ft container freights increased by 477% on the China-US route and by 243% on the China–Europe route [44].

COVID-19 has led to disruptions in global supply chain connections, causing major problems in international trade [45]. In the study by Cengiz & Turan [46] carried out with the participation of experts from 21 countries to examine the effect of COVID-19 on maritime transport, it was determined that BCO companies faced major challenges in their import processes. Particularly in the case of the uncertainty of delivery times, companies are unable to make plans about raw materials and production and have to deal with unanticipated financial losses in supply chain processes, such as packaging, storage and distribution. In one study, it was reported that 94% of the companies on the Fortune 1000 have experienced major problems in their supply chain connections due to issues of uncertainty in supply chain operations caused by the pandemic [47]. One of the main methods applied by maritime companies to address the delays in both inland transportation and in-port transfers of export cargoes has been to reduce the "Free Time" of containers. Some BCOs choose to leave their cargoes at the terminals due to short free times and higher expiration fees, such as demurrage and detention costs [48].

### Solution strategies developed during the COVID-19 era

To address the problems in maritime transportation, many maritime lines have chosen to increase the end-to-end logistics service networks that they offer to foreign trade companies through horizontal and vertical integration expansion [41]. Because the container transport chain comprises several different business processes; therefore, to move a container from one point to another, many independent parties must continuously communicate with each other. For example, avocado shipment in containers between Mombasa and Rotterdam encompasses 30 independent parties and 100 employees in the process and conducted 200 information transfers [49]. Shipping lines provide logistical services to prevent communication gaps in the chain and direct communication with the shipper during all processes, including the shipment and delivery of the cargo to the customer (E2E), rather than just providing container transportation services. For example, A.P. Moller-Maersk, which has the world’s largest container-ship fleet, provides E2E logistical services, including major services, such as inland transport, warehousing, customs, and distribution, in addition to container-transportation service [50].

Due to the low level of demand for container transportation, containers that have already been booked are kept in warehouses for some period of time and bookings for the subsequent days increase the demand. Thus, ships cannot be available and often containers are rolled over. Many strategies have been developed by shipping lines to resolve this issue. The practice of shipping guarantee booking developed by Hapag-Lloyd is one of these strategies. Thanks to this practice, the company helps its customers who struggle against uncertainties in the supply chain by preventing container rollovers during the uncertainty period caused by COVID-19, especially during the high-volume raw material import period [51].

When empty containers cannot be supplied, purchasing or renting containers can be an important resolution to the container-shortage issue. In a recent study, Ko [21] has concluded that purchasing containers is much more cost-effective than renting. In addition, there are advantages to purchasing containers for the shipper, such as the elimination of demurrage and detention costs, which would normally be paid to the container shipping company because of excess free time.

Another method developed to overcome the insufficiency of empty containers is the inducement call. The demand uncertainty because of communication gaps is among the main reasons for the container shortage. For example, Maersk announced periodical blank sailing in services where the demands decreased because of COVID-19 and then decided to make an inducement call to the ports where there was sufficient demand for the ships operating within the canceled route [52]. Thanks to this, BCOs could continue their activities within regions of high demand. In this process, many methods have been tried in order to follow customer demands and eliminate communication deficiencies. Logistics information systems-based technologies were insufficient to meet the needs in this process. Information and communication technologies, which have provided digital visibility, must be redesigned with the technology of the autonomous age to provide resistance to the container-transportation chain against the difficulties caused by COVID-19. Information communication technologies redesigned with Industry 4.0, a new phase in the Industrial Revolution that provides digital visibility to managers of companies in the container-transportation chain and enables large data to be collected, transmitted, and processed at one point, presents real-time visibility and decision-making support by optimizing the entire transportation chain within the virtual environment [53]. Accordingly, shipping companies benefit from the block chain infrastructure, which is an important achievement of Industry 4.0, to achieve a continuous connection within the container-transportation chain during COVID-19 [54]. The Mediterranean Shipping Company (MSC) has launched the “electronic Bill of Lading” (eB/L) application in Wave BL with block chain technology to enable continuous communication among the parties in the chain [55]. Another important example within the scope of information and communication technologies is the “spot booking application”, which was developed before COVID-19 and launched by the A.P. Moller-Maersk Group, which offers a web-based solution to BCOs for global uncertainties during the pandemic period. This application is an information communication technology that allows BCOs to create a reservation record for a ship without contacting a company representative at the reservation stage [56].

## Methodology

In the multi-criteria decision model stage, it is important to determine criterion weights. Many different methods with unique characteristics can be used to determine criterion weights and similarly, there are numerous methods for determining alternatives. In the present study, the SWARA (Step-wise Weight Assessment Ratio Analysis) method was used to rank the criteria representing the problems, while the ARAS (Additive Ratio Assessment) method was used to rank the alternatives representing the solution methods. The primary reasons for choosing these methods were that the two methods are compatible with each other, are simple in terms of mathematical operations, and have high reliability and simple outputs [57]. The procedures related to both methods are described below.

### SWARA (step-wise weight assessment ratio analysis) method

SWARA is a decision-making method developed by Keršuliene et al. [58]. One of the advantages of this method, as compared to both simple and other multi-criteria decision methods, is that it provides the opportunity to obtain the same results with a limited number of mathematical operations [59]. For example, Erdoğan et al. [60] demonstrated that the same results for determining criterion weights as those achieved using the ANP (Analytic Network Process) can be achieved with the SWARA method using fewer mathematical operations. More specifically, it was shown in one case that while 67 binary comparisons were made in ANP, 9 comparisons were made in SWARA, with similar results. In a similar study carried out by Stanujkic et al. [61], SWARA and AHP (analytic hierarchy process) methods were compared, and it was determined that it was easier to calculate mathematical operations by making less comparisons with the SWARA method, as compared to the AHP method. The SWARA method is appropriate for solving the decision problem where the importance level of the criteria is known [62]. In addition, this method allows the experts who created the data set to freely express their feelings, thoughts, and opinions without being dependent on external factors. From the data obtained from sectoral experts, it is possible to solve the incompatibility problem and create rational decision models by examining the criterion weights [63]. Therefore, the decision hierarchy formed as a result of the data analysis is seen as the numerical equivalent of the experts’ experiences [58]. On the other hand, the main disadvantage of the SWARA method over other MCDM methods is that the calculation procedure does not include a procedure for determining the consistency of pairwise comparison [61].

Examination of the literature shows that the SWARA method has been used to come up with solutions to many decision-making problems. For example, in a study by Prajapati et al. [64],​the relative impact of barriers to reverse logistics implementation was evaluated using SWARA, while in a study by Agarwal et al. [65], the ranking of criteria related to the creation of barriers to human supply chain management was evaluated by using fuzzy SWARA to improve it. The SWARA method has also been applied to rank the problems caused by the lack of sustainable suppliers in the electronics industry [66]. Moreover, Yücenur and Ipekçi [67], in the model they proposed, ranked the criteria for a site selection problem by using SWARA for the first offshore current power generation facility planned to be established in Turkey, while Baç [68], who evaluated different smart card systems, used the SWARA method to determine the best alternative and the criteria weights in the decision model.

With the SWARA method, the weights are determined using the following six steps:

1. The decision problem is determined and the criteria related to the problem are defined. The defined criteria are classified as main and sub-criteria. A decision committee consisting of *k* decision makers(${DM}_{k}, k=\mathrm{1,2},\dots , K)$ is established.
2. Each decision maker (${DM}_{k}$) participating in the decision-making process evaluates the main criteria and sub-criteria determined for the problem among themselves based on his or her own experience and knowledge. Evaluated criteria are ranked in descending order of qualitative importance.
3. After each decision maker ranks the criteria, the relative importance levels of the criteria are determined and scores between 0 and 1 are assigned. The criterion considered to be the most important is given a score of 1; the others are scored in multiples of 5. The $t.$ criterion is then compared with the previous criterion (t − 1) to determine a ratio called the “relative importance of the mean value” and denoted by ${S}_{t}$. As an example, ${DM}_{1}{S}_{1}$ shows the mean value of the comparative weight between the 1st important criterion and the 2nd important criterion for the decision maker 1.
4. For each criterion, the coefficient of the criterion is calculated as shown in Eq. (1), the most important of which is *k*_*t*_ and assigned a 1.
  1 $${k}_{t}=\left\{\begin{array}{l} 1\quad if\,\, t=1\\ {S}_{t}+1 \quad if \,\, t> 1\end{array}\right.$$
5. The weight (*w*_t_) for each criterion is calculated as given in Eq. (2). The *w*_t_ coefficient of the most important criterion is assigned a 1.
  2 $${w}_{t}= \left\{\begin{array}{c}1\quad if\, t=1\\ \frac{{w}_{t-1}}{{k}_{t}}\quad if\, t>1\end{array}\right.$$
6. The final weights (*q*_*t*_) of the criteria with calculated weights (*w*_t_) are calculated as in Eq. (3).
  3 $$q_{t} = \frac{{w_{t} }}{{\sum {w_{t} } }}$$

To reduce the criterion weight determined by each decision maker to a single value, integration is performed by taking the arithmetic average of the calculated weight of each decision maker for the relevant criterion, after which the final criterion weight is obtained.

### ARAS (additive ratio assessment) method

The additive ratio assessment (ARAS) method was developed in 2010 to provide a new approach for resolving multi criteria decision-making issues [69]. This method was developed to calculate the utility ratios of the alternatives and to rank the most appropriate alternatives using simple mathematical calculations [70]. The procedures used for the mathematical calculations of the ARAS method are simpler than prominent MCDM methods, such as TOPSIS (Technique for Order of Preference by Similarity to Ideal Solution), VIKOR (VIse KriterijumsaOptimiz acija I Kompromisno Resenje), and PROMETHEE (Preference ranking organization method for enrichment evaluation) [71]. In MCDM methods, it is very important to use the utility function to evaluate the decision makers and to create the numerical equivalents of all combinations of alternatives. This is where the ARAS method has a major advantage, insofar as the alternatives in the decision hierarchy are ranked according to their utility function values [72] and compared with the utility function value of the optimal alternative determined by the decision maker. In other words, the utility function value in the ARAS method is equivalent to the relative effect of the values and weights of the alternatives. This method allows complex problems to be simplified and the most appropriate alternatives to be ranked with their utility ratios, without using different calculation tools [73]. To simplify, by determining the utility function value, the most appropriate alternatives can be ranked [69]. It is possible to determine this value by proving other independent conditions after finding the multi-attribute utility function and then creating multi-attribute utility functions [74]. This provides compatibility in many different sectors due to the fact that it yields accurate results in terms of determining easy, understandable, and most appropriate alternatives [58]. The ARAS method has been used in more than 95 scientific articles between 2010 and 2020 due to its advantages [73]. These articles used the ARAS method to determine everything from underground site selection for hydrogen storage [75] and calculation of sustainability indicators for renewable energy systems [58] to concept selection for load distribution [76], the most appropriate personnel selection [77] and accountant selection [78].

Resolving the decision-making issues using the ARAS method entails the following four steps:

1. After determining the alternatives and the weighted criteria, a decision matrix is created in which decision makers score the alternatives according to the criteria. The most distinctive feature of the ARAS method is that it creates optimal raw values in the initial decision matrix consisting of the optimal values of each criterion.

In the X decision matrix as shown in Eq. (4), *m* is as the number of alternatives and *n* is the number of criteria; *x*_ij_ is the performance value of *i*th alternative in the *j*th criterion, while *x*_0j_ is the optimal value of the *j*th criterion using Eq. (4).

4 $$X = \left[ {\begin{array}{*{20}c} {x_{01} } & \cdots & {x_{0j} } & \cdots & {x_{0n} } \\ \vdots & \ddots & \vdots & \ddots & \vdots \\ {x_{i1} } & \cdots & {x_{ij} } & \cdots & {x_{in} } \\ \vdots & \ddots & \vdots & \ddots & \vdots \\ {x_{m1} } & \cdots & {x_{mj} } & \cdots & {x_{mn} } \\ \end{array} } \right]; i = 0,1, \ldots ,m\quad j = 1,2 \ldots ,n$$

For the decision-making issue, when there is no information about the optimal value of the relevant criterion, the optimal value is calculated by using Eq. (5) when the criterion shows the utility attribute or using Eq. (6) when it shows the cost attribute.

5 $$\mathrm{Utility function}: {x}_{0j}=\underset{i}{\mathrm{max}}{x}_{ij}$$

6 $$\mathrm{Cost function}: {x}_{0j}=\underset{i}{\mathrm{min}}{x}_{ij}$$

1. When different scales and units are used for each criterion determined for the decision-making issue, the performance values are converted into a common unit for comparison. Using the ARAS method, the normalized matrix is shown as $\overline{X}$ and consists of $\overline{x}_{ij}$ values. The $\overline{x}_{ij}$ values are calculated as shown in Eq. (7) when they have a criterion utility attribute.
  7 $$\overline{x}_{ij} = \frac{{x_{ij} }}{{\sum\limits_{i = 0}^{m} {x_{ij} } }}$$

When ${\bar{x}}_{ij}$ values show the cost attribute, the cost function is converted to the utility function using Eq. (8) and the normalized value is calculated using Eq. (9).

8 $$x_{ij}^{*} = \frac{1}{{x_{ij} }}$$

9 $$\overline{x}_{ij} = \frac{{x_{ij}^{*} }}{{\sum\limits_{i = 0}^{m} {x_{ij}^{*} } }}$$

After calculating the normalized values of the criterion performance values, the normalized decision matrix $\overline{X}$ is obtained using Eq. (10).

10 $${\overline{\text{X}}} = \left[ {\begin{array}{*{20}c} {{\overline{\text{x}}}_{{{01}}} } & \cdots & {\overline{x}_{0j} } & \cdots & {\overline{x}_{0n} } \\ \vdots & \ddots & \vdots & \ddots & \vdots \\ {\overline{x}_{i1} } & \cdots & {\overline{x}_{ij} } & \cdots & {\overline{x}_{in} } \\ \vdots & \ddots & \vdots & \ddots & \vdots \\ {\overline{x}_{m1} } & \cdots & {\overline{x}_{mj} } & \cdots & {\overline{x}_{mn} } \\ \end{array} } \right]; i = 0,1, \ldots ,m j = 1,2 \ldots ,n$$

1. The weighted normalized decision matrix $\hat{X}$ is formed using the criterion weight determined in accordance with the opinions of the decision makers. The criterion weight satisfies the condition 0 < *w*_j_ < 1 and the sum is limited by Eq. (11).
  11 $$\sum_{j=1}^{n}{w}_{j}=1$$

The members of $\hat{X}$ weighted normalized decision matrix $\hat{x}_{ij}$ are obtained as shown in Eq. (12).

12 $$\hat{x}_{ij} = \overline{x}_{ij} \cdot w_{ij}$$

Weighted normalized decision matrix $\hat{X}$ is obtained in the matrix form given in Eq. (13).

13 $$\hat{X} = \left[ {\begin{array}{*{20}c} {{\hat{\text{x}}}_{{{01}}} } & \cdots & {\hat{x}_{0j} } & \cdots & {\hat{x}_{0n} } \\ \vdots & \ddots & \vdots & \ddots & \vdots \\ {{\hat{\text{x}}}_{{{\text{i1}}}} } & \cdots & {\hat{x}_{ij} } & \cdots & {{\hat{\text{x}}}_{{{\text{in}}}} } \\ \vdots & \ddots & \vdots & \ddots & \vdots \\ {{\hat{\text{x}}}_{{{\text{m1}}}} } & \cdots & {{\hat{\text{x}}}_{{{\text{mj}}}} } & \cdots & {{\hat{\text{x}}}_{{{\text{mn}}}} } \\ \end{array} } \right]; i = 0,1, \ldots ,m j = 1,2 \ldots ,n$$

4. The optimal function ${S}_{i}$ is calculated for each alternative determined in the decision-making issue, and the alternatives are evaluated according to the criteria. ${S}_{i}$ indicates the optimal function value of the *i*^th^ alternative, and the scores of the alternatives are obtained using Eq. (14).
  14 $${S}_{i}=\sum_{j=1}^{n}, i=\mathrm{0,1},\dots ,m$$

The value > ${S}_{i}$ calculated for each alternative indicates the most effective alternative for the decision-making issue. Using Eq. (15), ${K}_{i}$ utility ratios are calculated by proportioning ${S}_{i}$ values of the alternatives to the ${S}_{0}$ optimal function value.

15 $${K}_{i}=\frac{{S}_{i}}{{S}_{0}},i=\mathrm{0,1},\dots ,m$$

The effects of the alternatives on resolving the decision-making issue can be calculated using the ${K}_{i}$ utility ratios within the range of [0, 1]. The alternatives can be assessed by ranking the utility ratios. By using the ${K}_{i}$ ratios, the relative efficiency of the utility function values of the alternatives are calculated. Accordingly, the alternatives are then assessed by ordering the values.

### Practice process of the present study

**Fig. 1:**
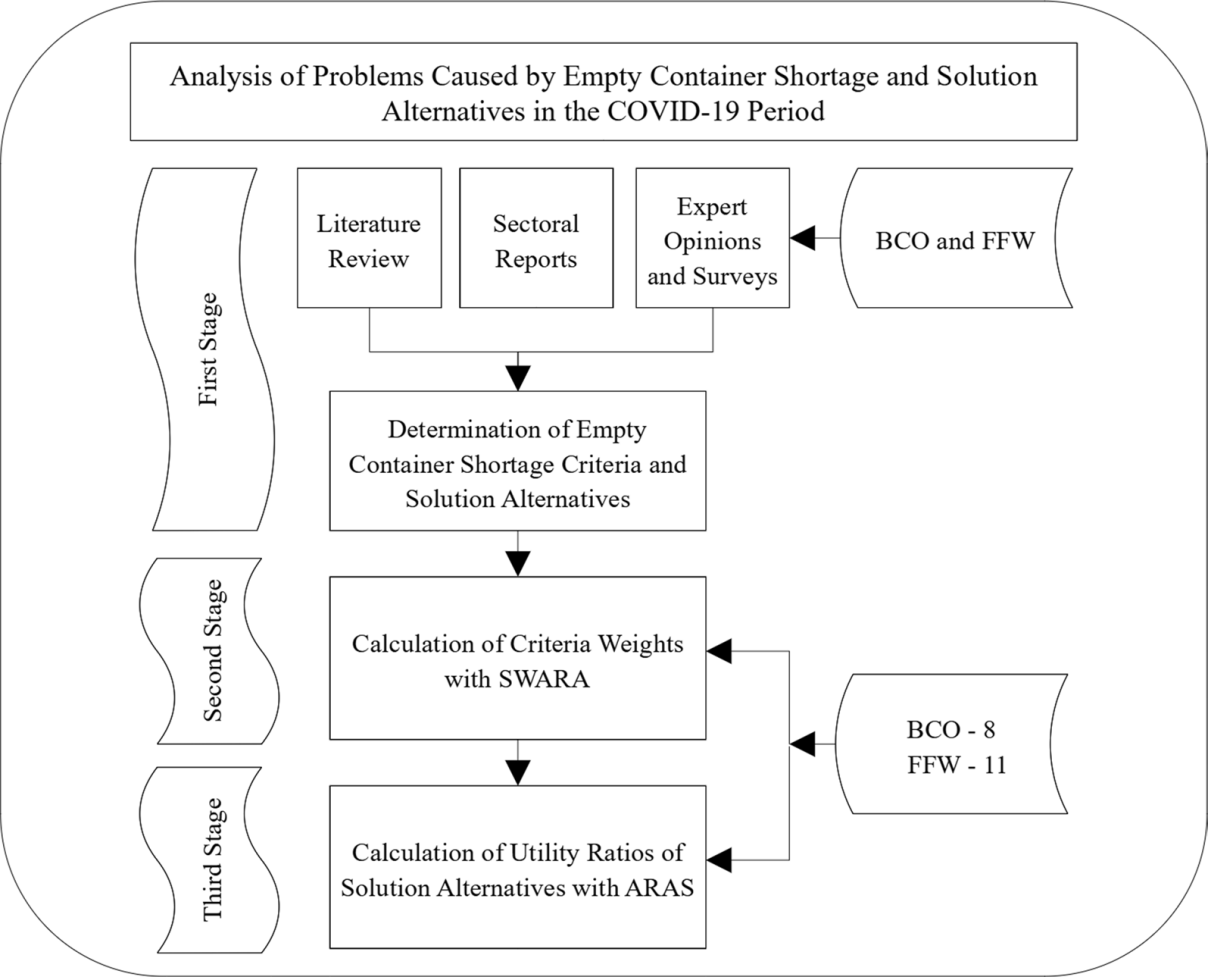
Flowchart of the study

In the first stage of the study, the literature was reviewed to determine the effects of the empty-container charge on container transportation and offer solutions, after which decision-making issues were identified using the opinions of 19 experts working in BCO and FFW companies that represent the demand for the container-transportation chain. In the second stage, criteria weights were calculated by evaluating the criteria determined by the experts. In the last stage, solutions were evaluated by experts using a range of 10–100 points according to the determined criteria, the optimum resolution proposal was calculated, and the study was concluded. The flowchart of the study is shown in Fig. 1.

### Instrument, sampling and data collection

The main criteria and subcriteria used as data collection protocols in the present study were constructed on the basis of studies reviewed in the literature, sectorial publications, and expert opinions. The criterion sampling technique was used to determine the group of experts to be involved in the study. Using this technique, the experts having sufficient experience and knowledge by which to evaluate the container-shortage issues were assessed using a set of questions (e.g., university graduation, sectorial experience, department, and position) and then included in the study. To evaluate the issues caused by the empty-container shortage on the demand (customer) side, the experts comprised 19 managers working in BCO and FFW companies. The sector representatives of the study group were interviewed through face-to-face and online applications.

To combine the sectorial experience with the theoretical knowledge to determine the expert group, a prerequisite was to have an associate degree/undergraduate education related to seaborne trade/transportation. Of all participants, 36.84% had completed their education in maritime business management, 31.58% in foreign trade, and 31.58% in international trade and logistics. In addition, 42.11% of the company representatives participating in the present study had 4–6 years of sectorial experience, 36.84% had 7–9 years, and 21.05% had 10–12 years (Table 1).

**Table 1:** Group of experts involved in the present study

	N	%
University department		
Maritime business management	7	36.84
Foreign trade	6	31.58
International trade and logistics	6	31.58
Total	19	100.00
Years of experience		
4–6	8	42.11
7–9	7	36.84
10–12	4	21.05
Total	19	100.00
Sector		
Freight forwarder (FFW)	11	57.89
Beneficial cargo owner (BCO)	8	42.11
Total	19	100.00

### Main criteria and sub criteria identified by the empty-container shortage

The precautionary strategies conducted to reduce the effects of COVID-19 have caused restrictive effects on global trade and major disruptions in labor productivity, production, and distribution processes and are considered in container transportation, the building block of global trade, as the container-shortage problem [79]. To reduce the negative effects of this problem on the container-transportation chain, many strategies have been developed during the COVID-19 pandemic. The issues within the container-transportation chain, the explanations of the criteria, and their abbreviations used in the calculation tables are presented in Table 2, while the suggestions to resolve these issues and their abbreviations are presented in the decision hierarchies in Table 3. In addition to the information obtained from the literature review, the main criteria and sub-criteria used in the decision hierarchy were determined, taking into account the suggestions of the experts working in the sector.

**Table 2:** Explanations and abbreviations of the main criteria and sub criteria determined in the present study

Main criteria–sub criteria	Abbrev	Definition
Container transportation cost increase	CTCI	Positioning the containers as empty without a return cargo to meet high empty container demands at the points of need causes shipping lines to deal with high transportation costs
Freight increase	FI	Freight rates have increased to the highest levels in recent years on trade routes where container shortage is considerable
Demurrage and detention cost increase	DDCI	Restriction and quarantine measures directly affect the workforce, causing port operations to slow down and containers to be kept in port areas or warehouses for long periods. This problem increases the demurrage and detention costs, which are known as free time violations that BCOs have to pay
Inland transportation cost increase	ITCI	Restriction policies developed during COVID-19 cause delayed port operations, resulting in port congestion. BCOs, who cannot receive service from the ports with heavy cargo and ship traffic, are forced to prefer ports at a distance for their export cargoes
Domestic inflation increase	DII	Compulsory storage of available cargoes for shipment due to lack of containers, has direct negative effects on the economic structures of countries
Supply chain uncertainty	SCU	All companies that import raw materials or require an empty container to ship export cargo (i.e., all companies that perform global trade activities at every stage of the supply chain) are negatively affected by the empty-container shortage
Holding available-to-promise loads	HAPL	As a result of the disruptions in business activities (insufficient number of drivers, slowdown of port and customs operations) experienced because of restrictive measures taken during COVID-19, containers are not transported to inland areas and are kept for long periods of time in warehouses at the port or surrounding areas
Freight rate uncertainty	FRU	Increased global demand, high increases in daily freighting rates, port congestion, and early launch of peak season surcharge tariffs by some shipping lines are important reasons that cause uncertainty in freight rates
Loss of companies’ competitive advantage	LCCA	The rapid increase in freight rates from increasing container shortages results in profits from the sales of many cargo groups remaining less than the transportation costs. BCOs struggling with this problem lose their competitive advantage against companies operating in the countries with high global trade
Long load shipment times	LLST	Even if the production capabilities of companies are at high levels, they cannot use their inventories when their chains of distribution do not work effectively; however, high demands of BCOs who want to replenish their stock levels that decreased because of the container shortage, cause congestion in ports and chains of distribution
Volume loss in container transportation	VLCT	The restrictions and quarantine practices at the ports cause prolongation of operation times in the port, but the ship frequency to ports in the regions where quarantine practices are high decreases. This causes periodic volume losses in container transportation
Increase in dry bulk cargo demands	IDBCD	Container shortage and cost increases in container transportation led to evaluating different alternatives for dry bulk cargo demand
Dry bulk freight increase	DBFI	The change in the demand increased spot freight rates for dry bulk carriers to their highest levels since 2008 and 2009
Intermodal transportation demand increase	ITDI	Container shortages cause the demand to shift from maritime trade, which provides low-cost freight transportation services, to more complex and specialized intermodal transportation
Highway freight increase	HFI	After unloading the cargo in the import zone, empty containers are kept in warehouses for some period of time until another demand. Accordingly, increased road freight rates increase container transportation costs in addition to storage costs
Blank sailing announcement increase	BSAI	When considering the reasons that the empty container cannot be quickly positioned, it was observed that shipping lines increase blank sailing announcements for ports located within regions heavily affected by the pandemic
Disruption of itineraries	DOI	The itinerary reliability is experiencing sharp decreases resulting from the pandemic
Loss of confidence to shipping companies	LCSC	As of 2020, the sharp decreases in the itinerary reliability and the problems from the container shortage considerably decreased the reliability levels for shipping lines
Unavailability in ships	UIS	When there is a decrease in the number of ships calling at ports, it becomes difficult to meet the increasing demand for container transportation
Rollover increases	RI	The demand burst for container transportation from decreased itineraries is not met by the small number of ships calling at the ports, and thus the majority of containers are often rolled over

**Table 3:** Explanations and abbreviations of the solution alternatives determined in the study

Resolution criteria	Abbrev	Definition
Shipper-owned containers	SOC	When empty containers cannot be supplied, an important solution would be to purchase them. Moreover, there are various advantages of purchasing containers for the shipper, such as the demurrage and detention costs, which have to be paid to the container shipping company because of excess free time, are eliminated
Inducement call	IC	By announcing periodic blank sailing within the services with decreased demands, shipping lines can direct ships along this service route to the ports with high demands. Thanks to the increase in the number of ships in the itinerary, BCOs can continue their activities within regions of high demands
Information communication technologies	ICT	COVID-19 has led to major changes in the structure of business models and global trade, emphasizing that information and communication technologies should be at the center of container transportation, not as an alternative method. Especially during this process, shipping lines benefit from technologies with block chain infrastructure to provide continuous connection in the container transport chain
Shipping guarantee booking	SGB	This application developed by shipping lines contributes to BCOs’ struggle against uncertainties in the supply chain by preventing container rollover during the uncertainty period because of COVID-19, especially during the high-volume raw-material import period
E2E delivery services	E2EDS	Demand uncertainty because of communication gaps is among the main reasons for the container shortage. Shipping lines provide logistical services to prevent communication gaps in the chain and provide direct communication with the shipper throughout all processes, including the shipment and delivery of the cargo to the customer (end-to-end; E2E), rather than just providing container transportation services

## Results

### Calculation of criteria ranking

The criteria weights were calculated using the SWARA method based on the knowledge and experience of each expert in the Decision Committee. In order not to prolong the study, only the findings of the first expert are presented in Table 4 as an example.

**Table 4:** Spreadsheet for first expert

Expert 1						
Main criteria	Queue	S_t_	k_t_	q_t_	w_t_	
CTCI	1	–	1	1	0.3833	
SCU	2	0.45	1.45	0.6897	0.2643	
VLCS	3	0.35	1.35	0.5109	0.1958	
BSAI	4	0.25	1.25	0.4087	0.1567	
			Total	2.6092		
*Container transportation cost increase*						
FI	1	–	1	1	0.3949	0.1514
DDCI	2	0.35	1.35	0.7407	0.2925	0.1121
DII	3	0.75	1.75	0.4233	0.1672	0.0641
ITCI	4	0.15	1.15	0.3681	0.1454	0.0557
			Total	2.5321		
*Supply chain uncertainty*						
HAPL	1	–	1	1	0.3510	0.0550
FRU	2	0.35	1.35	0.7407	0.2600	0.0407
LLST	3	0.25	1.25	0.5926	0.2080	0.0326
LCCA	4	0.15	1.15	0.5153	0.1809	0.0283
			Total	2.8486		
*Volume loss in container transport*						
ITDI	1	–	1	1	0.4332	0.0848
DBFI	2	0.75	1.35	0.5714	0.2476	0.0485
IDBCD	3	0.45	1.25	0.3941	0.1707	0.0334
HFI	4	0.15	1.15	0.3427	0.1485	0.0291
			Total	2.3082		
*Blank sailing announcement increase*						
UIS	1	–	1	1	0.4770	0.1261
DOI	2	0.85	1.25	0.5405	0.2578	0.0681
RI	3	0.75	1.15	0.3089	0.1473	0.0389
LCSC	4	0.25	1.15	0.2471	0.1179	0.0312
			Total	2.097		

The criteria weights were calculated in line with the opinions of experts working in BCO and FFW companies. The criterion weights of the decision committee were found by averaging the weights using the arithmetic mean operator in order for the weights of the calculated criteria to express the consensus opinion of the decision committee. The ranking of all criteria according to their weights and importance levels is given in Table 5.

**Table 5:** Decision committee weights of all criteria

Main criteria	Ranking	Weights
CTCI	(1)	0.3504
SCU	(2)	0.2397
VLCS	(3)	0.2210
BSAI	(4)	0.1887
*Container transportation cost increase*		
FI	(1)	0.1322
DDCI	(2)	0.0786
DII	(3)	0.0758
ITCI	(4)	0.0636
*Supply chain uncertainty*		
HAPL	(1)	0.0646
FRU	(2)	0.0591
LLST	(3)	0.0588
LCCA	(4)	0.0572
*Volume loss in container transportation*		
ITDI	(1)	0.0588
DBFI	(2)	0.0553
IDBCD	(3)	0.0552
HFI	(4)	0.0515
*Blank sailing announcement increase*		
UIS	(1)	0.0569
DOI	(2)	0.0511
RI	(3)	0.0462
LCSC	(4)	0.0344

The main criteria for the empty container shortage were ranked as follows based on their importance levels: CTCI (35.04%), SCU (24.98%), VLCT (22.11%), and BSAI (18.88%). The main criteria were then ranked according to their importance as follows: NI (13.22%) in CTCI, HAPL (6.46%) in SCU, ITDI (5.89%) in VLCT, and UIS (5.70%) in BSAI. When the general rankings were created according to the importance levels among the criteria, the first five subcriteria were as follows: NI (13.22%), DDCI (7.86%), DII (7.58%), HAPL (6.46), and ITCI (6.36%).

### Calculation of alternative ranking

To determine the most appropriate method among the suggested alternatives, the evaluations between 10 and 100 points of the experts participating in the study were taken as a reference. A decision matrix was created using the geometric mean to express the common opinion. The results of the calculated group decision matrix are shown in Table 6, while the results of the group decision matrix weighted for the alternatives are shown in Table 7.

**Table 6:** Group decision matrix for alternatives

	Container transportation cost increase (CTCI)			
Weights	0.3503	0.2397	0.2210	0.1887
Sub criteria	FI	DDCI	ITCI	DII
Optimal values	56.6442	47.1252	30.8617	33.6621
SOC	56.6442	47.1252	25.2376	32.5803
IC	44.4703	41.3827	26.3886	33.6621
ICT	44.5072	34.4911	29.0413	31.4131
SGB	52.6801	46.9664	30.8617	30.9411
E2EDS	33.5620	34.0236	29.7509	24.9430
Total	231.8641	203.9891	141.2804	153.5398

	Supply chain uncertainty (SCU)			
Weights	0.1322	0.0786	0.0636	0.0758
Sub criteria	LCCA	FRU	HAPL	LLST
Optimal values	77.5595	56.4845	56.5221	59.1488
SOC	53.7686	54.6967	52.1422	47.4304
IC	55.1970	39.8257	45.9136	54.7510
ICT	68.2828	46.6060	56.5221	47.4498
SGB	77.5595	56.4845	50.8902	59.1488
E2EDS	34.1473	32.4698	34.6452	37.9423
Total	288.9555	230.0829	240.1136	246.7224

	Volume loss in container transportation (VLCT)			
Weights	0.0572	0.0591	0.0646	0.0588
Sub criteria	ITDI	IDBCD	DBFI	HFI
Optimal values	68.6913	61.2956	57.2613	48.6189
SOC	54.2547	54.3676	51.5894	39.4703
IC	55.4120	49.1203	43.9511	48.1737
ICT	68.6913	61.2956	57.2613	48.6189
SGB	64.2280	51.9102	53.6213	44.4890
E2EDS	26.2644	26.7975	31.6554	29.0847
Total	268.8507	243.4914	238.0787	209.8368
*Blank sailing announcement increase (BSAI)*				
Weights	0.0588	0.0552	0.0553	0.0515
Sub criteria	UIS	LCSC	DOI	RI
Optimal values	51.9071	52.6893	60.0266	57.2747
SOC	32.3597	33.6263	38.3663	45.0151
IC	46.6866	49.8301	54.1732	57.2747
ICT	51.9071	52.2278	60.0266	54.8104
SGB	42.0872	52.6893	54.4254	54.1832
E2EDS	25.9920	29.6552	27.8526	28.0962
Total	199.0329	218.0290	234.8442	239.3798

**Table 7:** Group decision matrix weighted for the alternatives

	Container transportation cost increase (CTCI)			
Weights	0.3503	0.2397	0.2210	0.1887
Sub criteria	FI	DDCI	ITCI	DII
Optimal Values	0.0855	0.0553	0.048	0.0413
SOC	0.0855	0.0553	0.039	0.0400
IC	0.0671	0.0486	0.0412	0.0413
ICT	0.0672	0.0405	0.0454	0.0386
SGB	0.0796	0.0552	0.0482	0.0380
E2EDS	0.0507	0.0399	0.0465	0.0306

	Supply chain uncertainty (SCU)			
Weights	0.1322	0.0786	0.0636	0.0758
Sub criteria	LCCA	FRU	HAPL	LLST
Optimal values	0.0354	0.0193	0.0149	0.0181
SOC	0.0246	0.0186	0.0138	0.0145
IC	0.0252	0.0136	0.0121	0.0168
ICT	0.0312	0.0159	0.0149	0.0145
SGB	0.0354	0.0193	0.0134	0.0181
E2EDS	0.0156	0.0110	0.0091	0.0116

	Volume loss in container transportation (VLCT)			
Weights	0.0572	0.0591	0.0646	0.0588
Sub criteria	ITDI	IDBCD	DBFI	HFI
Optimal Values	0.0146	0.0148	0.0155	0.0136
SOC	0.0115	0.0131	0.0140	0.0110
IC	0.0117	0.0119	0.0119	0.0135
ICT	0.0146	0.0148	0.0155	0.0136
SGB	0.0136	0.0126	0.0145	0.0124
E2EDS	0.0055	0.0065	0.0085	0.0081
*Blank sailing announcement increase (BSAI)*				
Weights	0.0588	0.0552	0.0553	0.0515
Sub criteria	UIS	LCSC	DOI	RI
Optimal values	0.0153	0.0133	0.0141	0.012
SOC	0.0095	0.0085	0.0090	0.0096
IC	0.0138	0.0126	0.0127	0.0123
ICT	0.0153	0.0132	0.0141	0.0118
SGB	0.0124	0.0133	0.0128	0.0116
E2EDS	0.0076	0.0075	0.0065	0.0060

The criteria weights calculated using SWARA and the weighted group decision matrix are shown in Table 7.

The optimum function, utility ratios, and resolution rankings calculated for the demand sector are presented in Table 8. When examining the findings, SGB was determined to be the most beneficial alternative with a 95.08% utility ratio among all alternatives proposed for the empty container shortage. The utility ratios of other resolution alternatives suggested in the study were ICT (88.28%), SOC (87.60%), IC (84.88%), and E2EDS (62.93%).

**Table 8:** Utility ratios of alternatives

Alternatives	Si	Ki	%Ki	Rank
Optimal values	0.4325	–	–	–
SOC	0.3788	0.8760	87.6044	3
IC	0.3670	0.8487	84.8777	4
ICT	0.3818	0.8828	88.2846	2
SGB	0.4112	0.9507	95.0792	1
E2EDS	0.2721	0.6292	62.9281	5

## Discussion

Considering the importance of identifying the problems experienced in the container transport chain that involves many global-scale companies and examining the solution strategies developed for these problems, the present study examines the impact of container shortages, which have intensified due to the worldwide spread of COVID-19, on the commercial activities of BCO and FFW businesses, which represent the demand side of container transportation. The experts who served as the data source of the study evaluated the alternatives developed for the solution to the container shortage problem, and a decision matrix was created to rank the most appropriate solution methods.

The criteria determined in this study to rank the problems causing empty container shortages during the COVID-19 period are compatible with those reported in the literature [32, 35, 42]. Moreover, the criteria and alternatives created by considering the literature and the opinions of experts and sectorial broadcasting organizations represent the problem and solution methods that were examined within the scope of the study [26, 33, 41, 46, 80].

According to the results of the study, the most appropriate method to solve the empty container shortage problem is the the use of a shipping guarantee booking application. This solution is considered the best by experts and is also the most preferred solution in the maritime sector [51, 81]. This is because the method contributes significantly to reducing storage costs, which are viewed as a substantial financial burden for BCOs, and to eliminating the unavailability problem of free space on container ships [26, 35].

The second most appropriate method identified from the results of this study is the use of information communication technologies. COVID-19 has led to major changes in business models and in the structure of global trade. These changes indicate that information and communication technologies must play a central role in container transportation and not be viewed simply as an alternative method [82]. This area of supply chain operations needs to be redesigned with the technology of the autonomous age to strengthen container transportation against the harsh conditions caused by COVID-19. The world’s largest shipping companies have established horizontally integrated collaborations with companies that deliver digital services in order to provide end-to-end communication in the container transport chain for BCOs [55].

The environment of uncertainty caused by container shortages has forced companies to deal with unforeseen financial losses [35]. As container shortages result in the available-to-promise products being kept in warehouses for long periods [83], BCOs operating in different sectors, such as retail, food, manufacturing, and industry, have started to purchase containers [84]. In this study, the shipper-owned container method developed by BCOs was determined to be the third most appropriate method for dealing with the container shortage problem.

Inducement calls were found to be the fourth most appropriate method for solving the container shortage problem. One of the key problems seen during this process is that available-to-promise cargoes are kept at the ports until the next port of call due to the rollover of containers [35]. One of the important solution methods that reduces the waiting time of cargoes at ports is the use of inducement calls, but this means additional ports of call [85]. Moreover, this solution method is directly related to the predictability/measurability of the demand for container transportation, as maritime companies can perform an inducement call only when there is sufficient demand in the region [52].

Lastly, the fifth most appropriate method for addressing the container shortage problem is the application of E2E Delivery Services. The provision of end-to-end services, rather than just port-to-port services, by maritime companies for all export–import processes likely contributes to providing BCOs the ability to better manage their reservations and thus eliminate the uncertainty in demand [86]. When the uncertainty in demand is eliminated, blank sailing or port of call cancellations will likely decrease and thereby help to overcome the container shortage problem [18]. This result of the study is compatible with that reported by a study demonstrating that providing end-to-end logistics services qualifies as one of the appropriate methods for solving the problems caused by COVID-19 [41].

## Conclusion

In the present study, the issues caused by the empty-container shortage were determined as 4 main criteria, and the solutions were determined as 5 alternatives. The input obtained from 19 managers and experts was analyzed using a hybrid method of SWARA and ARAS. The results of study indicated that ranking of the problems caused by the lack of containers in the COVID-19 period; (1) cost increase in container transportation, (2) supply chain uncertainty, (3) volume loss in container transportation, (4) increased in blank sailing. In addition, the most suitable solutions; (1) shipping guarantee booking, (2) information communication technologies, (3) shipper owned container, (4) inducement calls, (5) E2E delivery services. We suggest that the results of the present study would provide significant new information to the literature and a resource for container-transportation stakeholders because they address the empty-container shortage and the methods by which to resolve this issue, which has been considerable as a result of the negative effects of COVID-19.

## Recommendations for the industry

Collaboration among the parties involved in the supply chain, such as BCO, FFW, shipping lines, and container terminal operators, can reduce the storage time of empty containers, efficiently use the existing capacity, and reduce congestion in the land and sea transportation lines of ports. Despite huge increases in the freight rates for container transportation, there is no decrease in the astronomical prices of spot demand and long-term chartering of ships, which indicates that this crisis will most likely continue. Within this period, it is considered that the establishment of long-term contracted agreements between shipping lines and BCOs and FFWs will contribute to both reducing spot rates and eliminating the uncertainty of regional demands. In addition, it will be considerably more important to establish data transparency and develop holistic and inclusive methods by which to reduce the devastating impact of the empty-container shortage, which has increased in severity on the global trade industry because of COVID-19. During this crisis, web-based optimization of all processes in the container transport chain, real-time monitoring of all data by the companies in the chain, and quick interventions when necessary would contribute to the formation of sustainable container transport chains in which empty containers are used more efficiently.

## Data Availability

Not applicable.

## Abbreviations

DM_k_: Decision maker
G_j_: Final weights of the criterion
$${\mathrm{K}}_{i}$$: Utility ratio
k_t_: Coefficient of the criterion
M: Number of alternative
n: Number of criteria
q_t_: Final weights of the criteria
Si: Optimal function
S_t_: Relative importance of the mean value
w_j_: Weight of the criterion (ARAS)
w_t_: Weight of the criterion (SWARA)
X_0j_: Optimal value of the jth criterion
X_ij_: Performance value of *i*th in the *j*th
$\overline{X}$: Normalized decision matrix
$\hat{X}$: Weighted normalized decision matrix
